# Editorial: Clinical application of psychiatric assessment and treatment in Psychosomatic diseases

**DOI:** 10.3389/fpsyg.2023.1253286

**Published:** 2023-08-01

**Authors:** Yujun Gao, Yiding Han, Jian Xu, Xiangjun Tang, Haohao Yan

**Affiliations:** ^1^Department of Psychiatry, Renmin Hospital, Wuhan University, Wuhan, China; ^2^Department of Psychiatry, The Second Xiangya Hospital, Central South University, Changsha, China; ^3^Hubei Minzu University, Enshi, Hubei, China; ^4^Neurosurgery of Zhou Lab, Yale University, New Haven, CT, United States; ^5^Department of Psychiatry, National Clinical Research Center for Mental Disorders, and National Center for Mental Disorders, The Second Xiangya Hospital of Central South University, Changsha, China

**Keywords:** Psychosomatic diseases, medication, psychotherapy, physical therapy, psychological assessment, fMRI

## Introduction

Psychosomatic diseases are widely regarded as a result of the complex interplay between both psychological factors and physiological conditions. As such, in the field of healthcare the diagnosis and treatment of such disorders have become a significant challenge, which would necessitate collaborations among multiple disciplines and professions (Zhong et al., 2023b), as well as close cooperation among physicians, psychologists, and other healthcare professionals (Settineri et al., 2019).

This Research Topic presents a collection of 10 original articles, comprising seven studies utilizing psychological scales and psychotherapy in patients with Psychosomatic diseases and three studies investigating the neuroimaging mechanisms underlying mood/cognitive function using resting-state functional magnetic resonance imaging and psychological scales. Among the seven studies on psychological scales and therapy, four primarily focused on assessing the disease status of Psychosomatic diseases encompassing substance use disorder (SUD), breast cancer, orofacial pain, and infertility; two studies evaluated treatment efficacy for bipolar disorder (BD) and Psychosomatic diseases; and one study explored psychotherapy for chronic pelvic pain. Among the three studies on the neuroimaging mechanism, two studies examined major depressive disorder (MDD); and one study investigated generalized anxiety disorder (GAD).

## Psychological scales and psychotherapy

Psychological assessment involves the collection of psychological information from individuals using various measurement tools and techniques to understand their cognitive abilities, emotional states, and behavioral manifestations (Grassi et al., 2014; Figueiredo-Ferraz et al., 2021). It is commonly used in clinical practice for diagnosing psychosomatic disorders, assessing their severity, and developing personalized intervention plans and treatment strategies. Two studies utilized psychological scales to evaluate disease severity and assist diagnosis. Huang G. et al. employed neuroimaging techniques along with Natural History Interview (NHI) and Barratt Impulsiveness Scale (BIS-11) to study the cognitive performance and neurofunctional impairments related to psychiatric conditions in methamphetamine (MA) abusers. Their research revealed a correlation between attentional bias in MA addicts and the N200 component, which can be used to detect psychiatric factors in abstinent MA abusers. Li et al. utilized the Somatic Symptom Disorder B Criteria Scale (SSD-12), Whiteley Index-8 (WI-8), and other measures to quantitatively assess patients' perceptions and coping strategies related to bodily discomfort, as well as the distress level of somatic symptoms. They also employed the Fear of Cancer Recurrence-4 (FCR-4) and Functional Assessment of Cancer Therapy-Breast (FACT-B) scales to evaluate the magnitude of fear of cancer recurrence and the quality of life in breast cancer patients. These assessments can help breast cancer patients understand their psychological factors and improve their quality of life.

Two studies used psychological scales to assess treatment efficacy. Schmidt et al. evaluated the effectiveness of biofeedback therapy using anonymous quantitative self-report questionnaires and qualitative semi-structured interviews. Jing et al. utilized efficacy scales to assess the treatment outcomes of bipolar disorder.

Additionally, international standardized psychological assessment scales need to be culturally adapted to be more scientifically applicable to the local population (Phillips et al., 1991; Shi et al., 2017). Ou-Yang et al. developed the Chinese version of the Biopsychosocial Impact Measurement-Short Form (BPIm-S), which demonstrated good psychometric quality and can be used to assess functional limitations and psychosocial distress in patients with orofacial pain in China. Mubashir et al. developed the Social Comparison Scale (SCS) and Submissive Behavior Scale (SBS), which exhibited acceptable psychometric properties in Pakistani women with primary infertility, as confirmed by confirmatory factor analysis with good model fit indices.

Psychological therapy, by modulating the bio-psycho-social factors, offers a new perspective on appropriate treatment for mind-body disorders (Hilbert et al., 2019). Huang J. et al. reported a case of chronic pelvic pain syndrome in which the patient achieved effective relief through a combination of medication and psychological therapy.

## Psychological scales and brain imaging

Neuroimaging techniques combined with psychological scales, have been extensively employed in investigating structural and functional brain disorders (Gao et al., 2022, 2023; Wang et al., 2022; Zhong et al., 2023a). Meng et al. found that abnormal connectivity patterns were observed in the left middle temporal gyrus in GAD, underscoring the significance of GAD pathophysiology. Zhou et al. found that adolescent MDD with a history of suicidal attempts exhibited reductions in the amplitude of low-frequency fluctuations in the bilateral medial superior frontal gyrus and bilateral precuneus, potentially serving as indicators of MDD and suicidal attempts. Additionally, Wang et al. discovered that decreased regional homogeneity in the salience network may contribute to cognitive impairments in patients with MDD.

## Summary

Psychological scale assessment involves expertise from various fields, including medicine, psychology, and sociology. These assessment scales can be combined with multiple research techniques to study psychosomatic disorders. The treatment of psychosomatic disorders requires the integration of knowledge from medicine, psychology, and sociology, among other disciplines. We advocate for enhanced interdisciplinary collaboration among experts in the medical, psychological, and sociological fields to collectively address the challenges posed by psychosomatic disorders and provide patients with improved assessment, treatment, and management approaches. Interdisciplinary collaboration can also facilitate prevention and early intervention of psychosomatic disorders, thereby reducing incidence rates and minimizing long-term negative impacts.

## Author contributions

YG and HY reviewed all articles, summarized individual studies' findings, and drafted the manuscript. All other editors participated in editing articles and reviewed and had access to the manuscript. All authors contributed to the article and approved the submitted version.

## References

[B1] Figueiredo-Ferraz H. Gil-Monte P. R. Grau-Alberola E. Ribeiro do Couto B. (2021). The mediator role of feelings of guilt in the process of burnout and psychosomatic disorders: a cross-cultural study. Front. Psychol. 12, 751211. 10.3389/fpsyg.2021.751211 35027899 PMC8748256

[B2] Gao Y. Guo X. Zhong Y. Liu X. Tian S. Deng J.. (2023). Decreased dorsal attention network homogeneity as a potential neuroimaging biomarker for major depressive disorder. J. Affect. Disord. 2023, S0165032723004330. 10.1016/j.jad.03080 36990286

[B3] Gao Y. Tong X. Hu J. Huang H. Guo T. Wang G.. (2022). Decreased resting-state neural signal in the left angular gyrus as a potential neuroimaging biomarker of schizophrenia: an amplitude of low-frequency fluctuation and support vector machine analysis. Front. Psychiatry 13, 949512. 10.3389/fpsyt.2022.949512 36090354 PMC9452648

[B4] Grassi L. Caruso R. Sabato S. Massarenti S. Nanni M. G. (2014). The UniFe psychiatry working group coauthors null. Psychosocial screening and assessment in oncology and palliative care settings. Front. Psychol. 5, 1485. 10.3389/fpsyg.2014.01485 25709584 PMC4285729

[B5] Hilbert A. Petroff D. Herpertz S. Pietrowsky R. Tuschen-Caffier B. Vocks S.. (2019). Meta-analysis of the efficacy of psychological and medical treatments for binge-eating disorder. J. Consult. Clin. Psychol. 87, 91–105. 10.1037/ccp0000358 30570304

[B6] Phillips M. R. Xiong W. Wang R. W. Gao Y. H. Wang X. Q. Zhang N. P.. (1991). Reliability and validity of the Chinese versions of the scales for assessment of positive and negative symptoms. Acta Psychiatr. Scand. 84, 364–370. 10.1111/j.1600-0447.1991.tb03161.x 1746289

[B7] Settineri S. Frisone F. Alibrandi A. Merlo E. M. (2019). Emotional suppression and oneiric expression in psychosomatic disorders: early manifestations in emerging adulthood and young patients. Front. Psychol. 10, 1897. 10.3389/fpsyg.2019.01897 31481915 PMC6710394

[B8] Shi C. Wang G. Tian F. Han X. Sha S. Xing X.. (2017). Reliability and validity of Chinese version of perceived deficits questionnaire for depression in patients with MDD. Psychiatry Res. 252, 319–324. 10.1016/j.psychres.03021 28314227

[B9] Wang Q. Gao Y. Zhang Y. Wang X. Li X. Lin H.. (2022). Decreased degree centrality values as a potential neuroimaging biomarker for migraine: a resting-state functional magnetic resonance imaging study and support vector machine analysis. Front. Neurol. 13, 1105592. 10.3389/fneur.2022.1105592 36793799 PMC9922777

[B10] Zhong Y. Chen Y. Zhou Y. Lyu Y-. A-. H. Yin J-. J. Gao Y.. (2023a). The Artificial intelligence large language models and neuropsychiatry practice and research ethic. Asian J. Psychiatry 84, 103577. 10.1016/j.ajp.2023.103577 37019020

[B11] Zhong Y. Huang J. Zhang W. Li S. Gao Y. (2023b). Addressing psychosomatic issues after lifting the COVID-19 policy in China: a wake-up call. Asian J. Psychiatry 82, 103517. 10.1016/j.ajp.2023.103517 36791610 PMC9918320

